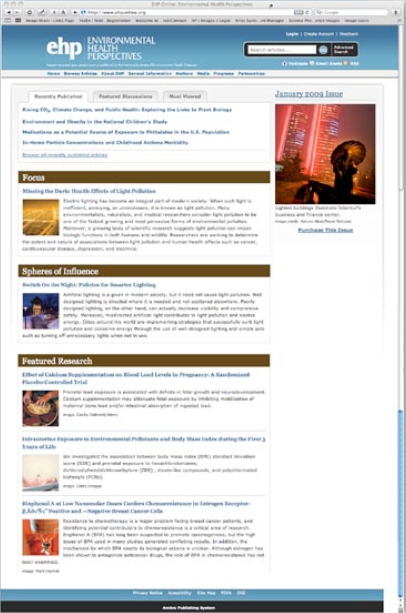# Notes from the Editor

**DOI:** 10.1289/ehp.117-2737030

**Published:** 2009-09

## Commentaries in EHP

Important advances in science often result from research that is novel or contrary to accepted paradigms. Often, the existing literature may be incomplete or inconsistent. Unfortunately, it is extremely difficult to review this type of work objectively—if we reject such papers we may be suppressing important ideas or concepts, but if we accept them we may be promoting “junk science.” The problem is exacerbated when the work in question is strongly associated with an individual, because it can be difficult, if not impossible, to separate the idea from the person. Also, in the absence of an objective basis or precedent for judgment, arguments “for” and “against” can become increasingly polarized to the point where there seems to be no middle ground between the two sides.

Like many journals, *EHP* has a section devoted to Commentaries that are intended to “present information and insight on a particular topic” [see *EHP*’s Instructions to Authors available online (http://www.ehponline.org/docs/admin/ita.html)]. *EHP* Commentaries therefore provide an effective and highly visible forum for discourse on new ideas and emerging issues. On rare occasions, Commentaries also serve as a platform for airing opposing sides of an argument. Such is the case with two Commentaries in this month’s issue (Mushak, p. 1333, and Calabrese, p. 1339).

We believe *EHP* Commentaries can advance environmental health by promoting open and constructive discussions about controversial topics and ideas. We reserve the right, however, to reject without review Commentaries we view as too polemic or personal in nature. We also reserve the right to propose that Commentaries be reviewed as one side of a “point–counterpoint” debate. If the original author agrees, we would ask another author to address the opposite side of the argument; if both papers were accepted, we would publish them together, as with this month’s articles by Mushak and Calabrese. After paired Commentaries are published, any additional rebuttals and critiques will be considered for publication only as Correspondence and would need to be formatted accordingly.

We look forward to publishing Commentaries that help us achieve our goal of advancing environmental health. As always, we welcome your feedback.

## New Website

*EHP* is pleased to announce a significant redesign of the journal’s website starting in September. The new website utilizes an open source platform for publishing our news and research articles. In addition to advanced search capabilities, we have included the ability to browse articles by subject category and publication date. There are RSS feeds for newly published articles by date and subject category. The website also includes community features such as threaded discussions; ratings for news articles; user profiles; formal correction and retraction annotations by *EHP* staff; and TrackBack support. We have improved capability to link to related articles and issues and to access recently published, most viewed, and featured discussion material. The new website also includes new features for article citation, as well as PDF and XML downloads. *EHP* welcomes your feedback and suggestions as you begin to explore the new features and layout of the new website. We consider the website to be a dynamic feature of the journal and intend to institute modifications and updates as needed.

## Figures and Tables

**Figure:**